# Timing of the Brunhes-Matuyama transition constrained by U-series disequilibrium

**DOI:** 10.1038/s41598-019-42567-2

**Published:** 2019-04-15

**Authors:** Bassam Ghaleb, Christophe Falguères, Julie Carlut, Jean-Pierre Pozzi, Geoffroy Mahieux, Larbi Boudad, Louis Rousseau

**Affiliations:** 1 GEOTOP, Université du Québec à Montréal, Montréal, Canada; 2 UMR 7194, Département Homme et Environnement, Muséum national d’histoire naturelle, 75013 Paris, France; 3 Laboratoire de Géologie, École normale supérieure, CNRS, UMR 8538, PSL Research University, 75005 Paris, France; 4 Institut de Physique du Globe de Paris (IPGP), Université Sorbonne-Paris-Cité, Université Paris Diderot, CNRS UMR7154 Paris, France; 5 Université de Picardie Jules Verne, EA 7511, Amiens, 80000 France; 6 University of Moulay Ismaïl, Faculty of Sciences, Meknes, 50 000 Morocco

## Abstract

U-series disequilibrium measurements carried out on thermogenic travertine samples from a 12.6 m-long core and a 10 m-thick section from southeastern Morocco yielded finite ages ranging from 500 ka to the present-day, as well as two clusters determined to be older than 500 ka. The calculation of initial ^234^U/^238^U activity ratios in all samples younger than 500 ka shows high, reasonably constant values, with an average of 5.172 ± 0.520 (one standard deviation). Assuming that this value prevailed for periods older than 500 ka, we derived ages of up to approximately 1.2 Ma using the initial ^234^U excess decay. Our results indicate that the two older clusters have ages of 776 ± 14 ka for samples from between 8 and 10.1 m and 1173 ± 22 ka for deeper samples respectively. The palaeomagnetic record of the core shows normal polarity inclinations from the surface to around 9 m followed by reverse polarity inclination and antipodal declinations. The inversion is attributed to the Brunhes-Matuyama transition. ^234^U excess ages for the interval corresponding to the part of the core where the polarity inversion occurred are in the range of 735 ± 51 to 794 ± 54 ka, with an arithmetic mean value of 776 ± 14 ka for the B-M transition. This age is in good agreement with that determined previously using other dating methods.

## Introduction

The U-series method is widely used to date biogenic CaCO_3_ (corals, mollusk shells), as well inorganic CaCO_3_ precipitates (speleothems, travertine)^1,2^ and references in^3,4^. U-series ages are derived from the activity ratios of ^230^Th/^234^U or ^230^Th/^238^U and ^234^U/^238^U^5–7^. Most terrestrial waters are enriched in ^234^U^8^ (i.e., are characterized by an ^234^U excess), and if the initial value of this excess is known, alternative ages can be calculated from the measured ^234^U/^238^U versus the initial ^234^U/^238^U ratio (^234^U/^238^U _initial_)^9^.

**Figure 1:**
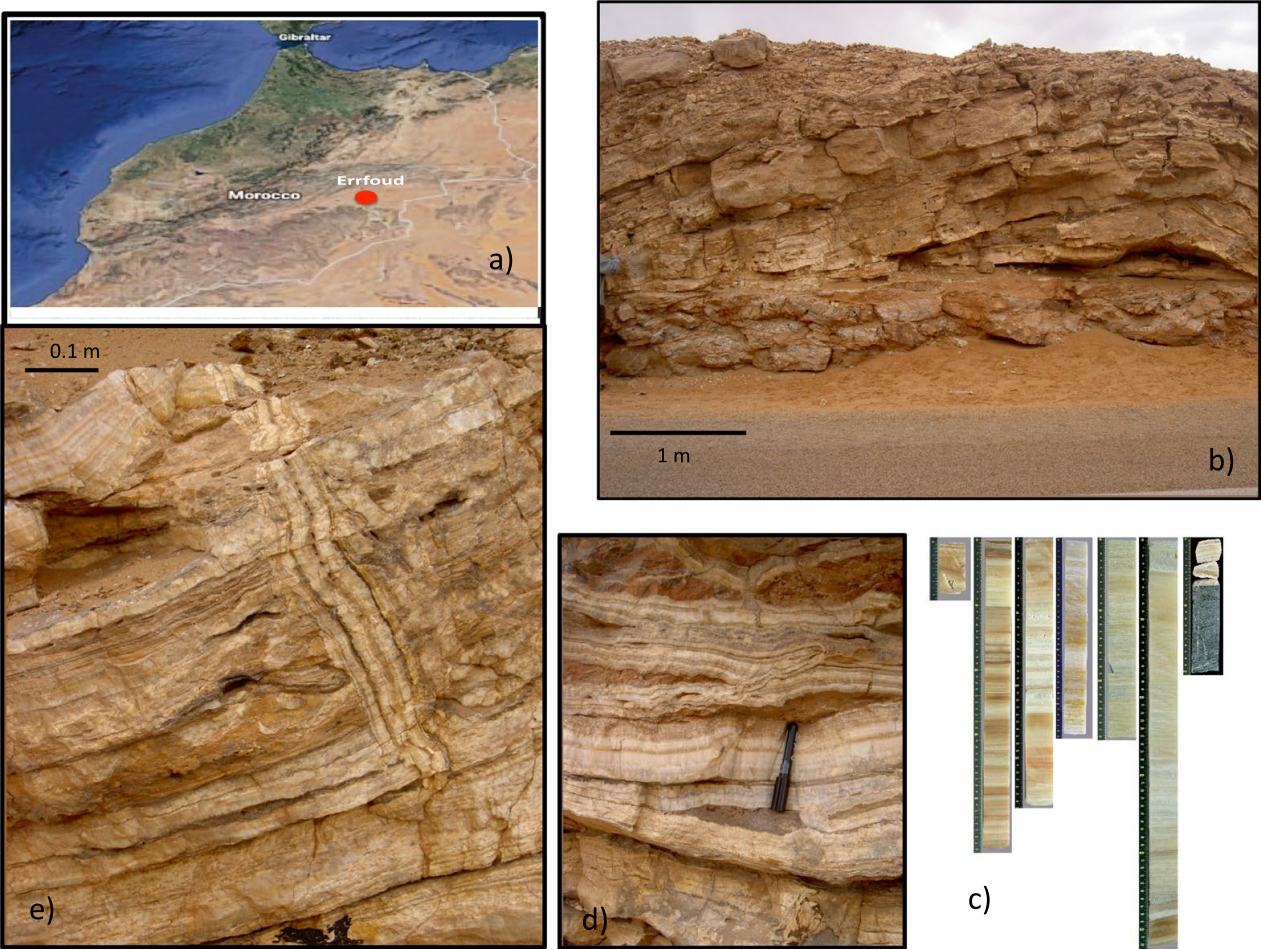
(**a**) The sampling location, Irdi, close to the city of Erfoud in southeastern Morocco (photo generated from https://earth.google.com/web/, version 9.2.80.4). (**b**,**d**,**e**) Different types of travertine samples encountered, showing anarchic growth with pure calcite layers alternating with clay levels, especially in the upper part. (**c**) Some sections of the 12.6 m-long extracted core, with the substratum at the base (left to right: top to bottom of the core; base at far right in the image).

Travertine deposits result from carbon dioxide-rich waters dissolving carbonate rocks at depth and then depositing calcium carbonate when pressure and CO_2_ decrease at the surface^10^. Travertine deposits are considered to be remnants of humid episodes, related to a positive hydrological budget^11^, and dating these deposits is therefore of great interest for climatic reconstruction. In the present study, the two U-series disequilibrium methods were combined to date hydrothermal thermogenic travertine deposits in southeastern Morocco, close to the city of Erfoud (Fig. 1a). The first method uses ^230^Th-^234^U-^238^U from samples younger than 500 ka and the second is based on the decay of initial ^234^U excess in samples aged between 500 and 1200 ka.

In addition, using this combination of the these two approaches, the present study extends the dating limit of U-series to approximately 1.2 Ma which gives us the ability to add new time constraints on the Brunhes-Matuyama magnetic reversal, which are independent of the K-Ar and ^40^Ar/^39^Ar radiometric dating methods or orbital tuning techniques that are usually used to date this transition.

## Materials and Methods

### Sampling

Samples were collected from a single section, approximately 10 meters long and 50 m wide, that was exposed as result of road construction, as well as from a 12.6 m-long core from Irdi, close to the city of Erfoud (Fig. 1b,c).

A few samples from the 10 × 50 m section were already dated^12^ via U-series using alpha spectrometry, and these results are reported along with the results obtained through the present study. The section is comprised of white, extremely clean CaCO_3_ layers alternating with brown or yellow layers with occasionally black beds. It is worth noting that the stratigraphic order was partially perturbed by recent pulses of hydrothermal activity that cut older travertine layers (Fig. 1d,e). As a result, stratigraphic layers cannot be directly correlated, and only chronological age dating can be used for this. A total of 12 samples from this section and nearby outcrops were analyzed by U-series. The core was also sampled for the carbonate facies. In the core, very clean CaCO_3_ layers were again found to alternate with darker brown layers. Sampling was determined by the lithology, and we focused on the clean CaCO_3_ layers, avoiding the discolored layers due to potential problems with detrital ^230^Th. A total of 26 samples were collected from the core (Table 1). For all U-series samples collected from the outcrop, zones showing stratigraphic perturbations were avoided and only layers following the laws of superposition were chosen for dating. In a similar way, sub-sampling from the core for U-series as well paleomagnetic plugs were all from visually undisturbed depths. For this reason the paleomagnetic and U-series samples have not been collected at regular intervals and any potentially perturbated areas have been avoided.

**Table 1:** U-series data and calculated ^230^Th-^234^U-^238^U and U/U_initial_ ages.

Location	Analytical technique	Depth (m)	^238^U ppm	±	(^234^U/^238^U)	±	(^230^Th/^238^U)	±	(^230^Th/U) age ka	Er.+	Er.−	(^234^U/^238^U)_0_	±	(U/U_0_) age ka	Er.+	Er.−
Road section	TIMS	Outcrop	2.134	0.013	4.711	0.017	1.060	0.008	26.932	0.338	0.335	5.005	0.018			
Road section	TIMS	Outcrop	2.030	0.013	4.829	0.020	1.031	0.008	25.412	0.325	0.322	5.114	0.020			
Road section	TIMS	Outcrop	6.096	0.040	2.290	0.011	2.786	0.023	515.124	96.071	57.110	6.533	0.770			
Road section	TIMS	Outcrop	2.136	0.014	5.070	0.018	0.521	0.005	11.670	0.150	0.149	5.206	0.018			
Road section	MC-ICP-MS	Outcrop	3.012	0.013	5.070	0.017	0.512	0.004	11.477	0.140	0.138	5.204	0.018			
Road section	MC-ICP-MS	Outcrop	3.054	0.011	5.055	0.015	0.516	0.004	11.593	0.134	0.133	5.190	0.015			
Road section	MC-ICP-MS	Outcrop	3.108	0.030	5.080	0.017	0.513	0.006	11.463	0.180	0.178	5.215	0.017			
Road section	α spectrometry	Outcrop	7.348	n.d.	2.773	0.005	2.984	0.077	261.554	22.905	19.681	4.713	0.206			
Road section	α spectrometry	Outcrop	2.252	n.d.	5.020	0.039	0.733	0.021	16.888	0.668	0.655	5.217	0.040			
Road section	α spectrometry	Outcrop	2.069	n.d.	4.637	0.041	1.150	0.048	29.992	1.712	1.662	4.959	0.044			
Road section	α spectrometry	Outcrop	1.900	n.d.	5.040	0.043	0.514	0.016	11.589	0.485	0.475	5.175	0.043			
Road section	α spectrometry	Outcrop	1.916	n.d.	5.503	0.100	0.963	0.063	20.479	1.895	1.804	5.771	0.103			
Core	TIMS	2.5	1.991	0.013	5.032	0.031	0.785	0.007	18.121	0.296	0.292	5.244	0.031			
Core	TIMS	3.6	1.909	0.014	5.068	0.029	0.730	0.009	16.640	0.326	0.322	5.264	0.029			
Core	TIMS	4.4	1.039	0.006	4.683	0.031	1.173	0.010	30.312	0.509	0.500	5.012	0.032			
Core	TIMS	4.8	2.653	0.012	2.381	0.015	2.811	0.024	398.307	40.322	31.056	5.257	0.272			
Core	TIMS	5.6	3.889	0.030	2.230	0.015	2.440	0.022	296.126	18.930	16.408	3.841	0.082			
Core	TIMS	8	2.109	0.009	1.442	0.008	1.754	0.017	n.d.	n.d.	n.d.			794.272	48.329	53.739
Core	MC-ICP-MS	8.09	2.914	0.001	1.466	0.003	1.659	0.011	643.793	n.d.	107.206			775.791	44.059	49.579
Core	MC-ICP-MS	8.92	2.827	0.009	1.461	0.003	1.644	0.011	578.565	176.650	75.129			779.534	44.081	49.601
Core	MC-ICP-MS	9.02	5.312	0.023	1.472	0.005	1.668	0.012	633.600	n.d.	117.537			770.887	45.383	50.880
Core	MC-ICP-MS	9.3	1.697	0.006	1.461	0.004	1.667	0.011	n.d.	n.d.	n.d.			779.073	44.466	49.979
Core	TIMS	9.3	1.572	0.009	1.443	0.006	1.648	0.013	n.d.	n.d.	n.d.			793.233	46.115	51.595
Core	MC-ICP-MS	9.5	1.374	0.005	1.523	0.005	1.749	0.012	748.977	n.d.	185.622			734.685	45.116	50.618
Core	TIMS	9.5	1.535	0.011	1.496	0.010	1.722	0.016	n.d.	n.d.	n.d.			753.560	48.794	54.186
Core	MC-ICP-MS	9.53	2.468	0.012	1.471	0.009	1.671	0.014	699.066	n.d.	188.557			771.862	48.140	53.557
Core	MC-ICP-MS	9.57	1.973	0.007	1.458	0.004	1.659	0.011	864.717	n.d.	268.411			781.815	44.345	49.861
Core	MC-ICP-MS	10	5.664	0.020	1.459	0.004	1.661	0.011	883.621	n.d.	291.562			781.164	44.799	50.307
Core	TIMS	10	1.117	0.007	1.458	0.006	1.656	0.013	750.475	n.d.	209.718			781.536	46.329	51.803
Core	MC-ICP-MS	10.1	1.571	0.006	1.455	0.004	1.652	0.011	761.578	n.d.	185.468			783.922	44.360	49.875
Core	TIMS	10.6	1.645	0.010	1.149	0.012	1.183	0.011	435.960	283.978	75.704			1179.479	71.728	74.894
Core	TIMS	11.15	7.789	0.045	1.174	0.006	1.261	0.010	n.d.	n.d.	n.d.			1125.141	53.120	58.291
Core	TIMS	11.5	1.831	0.015	1.178	0.006	1.259	0.022	n.d.	n.d.	n.d.			1116.678	53.232	58.397
Core	TIMS	12.3	1.711	0.009	1.131	0.007	1.168	0.011	485.095	n.d.	87.368			1225.640	62.363	66.745
Core	TIMS	12.4	0.960	0.006	1.171	0.006	1.199	0.009	392.544	58.195	38.423			1130.691	54.714	59.780
Core	MC-ICP-MS	12.52	0.810	0.003	1.133	0.005	1.208	0.014	n.d.	n.d.	n.d.			1219.197	54.596	59.670
Core	MC-ICP-MS	12.55	0.699	0.003	1.134	0.007	1.212	0.014	n.d.	n.d.	n.d.			1216.019	60.719	65.272
Core	MC-ICP-MS	12.6	0.957	0.004	1.141	0.005	1.198	0.010	633.671	n.d.	163.217			1197.528	55.260	60.287

### Analytical methods

#### U-series

Pieces of travertine ranging between 0.5 to 1 g were cut using a Dremel^TM^ diamond saw and washed with distilled water. The travertine samples were covered by desionized water and dissolved using clean distilled concentrated nitric acid in a Teflon beaker, into which weighed amounts of mixed ^233^U-^236^U-^229^Th spike was added and evaporated to dry at low temperature ~60 °C. Chemical extractions and purifications performed similarly to in^13^; briefly, U and Th were co-precipitated with Fe(OH)_3_. The precipitate was washed twice with water, and then dissolved in 6 N HCl. The U-Th separation was performed on a 2 ml volume of AG1X8 anionic resin. The Th fraction was recovered by elution with 6 N HCl and the U and Fe fractions by H_2_O. The U fraction was purified in a 0.2 ml volume of U-Teva (Elchrom industry^TM^) resin. The Fe was eluted with 3 N HNO_3_, and the U fraction with 0.02 N HNO_3_. Thorium purification was carried out on a 2 ml AG1X8 resin in 7 N HNO_3_ and eluted with 6 N HCl. After drying, a final purification step was performed on a 0.2 ml AG1X8 resin in 7 N HNO_3_, and Th was eluted with 6 N HCl. U-Th measurements were performed using a VG sector thermal ionisation mass spectrometer (TIMS) fitted with an electrostatic filter and a Daly ion counter. The U and Th fractions were deposited on a single zone-refined rhenium filament between two layers of colloidal graphite. U and Th isotopes were measured in peak jumping mode on the Daly ion counter.

A few samples from depths of greater than 8.1 m were analyzed by multi-collector inductively mass spectrometry, a Nu II instrument ^TM^ MC-ICP-MS, in order to achieve higher precision. The mass spectrometry analyses were performed at Geotop, Montreal^14^. For samples measured by alpha spectrometry at the IPH laboratory of Paris, the spike was ^232^U-^228^Th and the final U and Th fractions were plated on discs covered with an aluminum film and counted in EGG Ortec alpha detectors. The overall analytical reproducibility was estimated through replicate measurements of a coral from Mayo Island (Cabo Verde) that dates from last interglacial age (see supplementary materials). Precision is typically better than 1% for TIMS and MC-ICP-MS, and 3–5% at 2 σ levels for alpha spectrometry.

#### Paleomagnetism

Paleomagnetism applied to Quaternary speleothems and travertine formations has led to increased interest in relation to their potential as climate archives^15^. When such archives contain small amounts of ferromagnetic minerals, they can provide high-resolution records of the geomagnetic field^16,17^. Fifty discrete samples were taken from the core, from 1 to 12.6 meters depth. Samples were taken every 15 to 50 centimeters, perpendicular to the core length, using a 2.6 cm-diameter drill. The magnetization of these samples was measured using a horizontal 2 G cryogenic magnetometer at ENS Paris. Natural remanent magnetization (NRM) intensities vary from a few 10^−6^ Am^−1^ to 10^−3^ Am^−1^. Samples were demagnetized using alternating field (AF) up to 90 mT, and a characteristic direction was successfully determined for the majority of the samples using principal component analysis^18^. Thermal demagnetization has been tested for six pilot samples. Secondary magnetization is erased above 200 °C or a few mT. Thermal and AF demagnetization give reproducible results.

In addition 49 small plugs of 1.5 cm diameter (vol = 2.5 cm^3^) were sampled between 8.69 and 9.84 m with a sampling step of 2 to 2.5 cm in order to refine the critical interval revealed by the analysis of the 2.6 cm cores. These samples underwent stepwise alternating field (AF) demagnetization in 6–8 steps between the natural remanent magnetization (NRM) and 70 mT using a Shonstedt demagnetizer at IPGP, steps to higher values were not performed due to suspicion for lack of stability of the instrument. Magnetization was measured using a 2 G cryogenic magnetometer at IPGP. NRMs range between 5 10^−5^ to 1 10^−2^ Am^−1^. Orthogonal vector plots using Paleomac software^19^ were used for principal component analysis.

IRM acquisition curves were performed on the 2.5 and 1.5 cm plugs up to 2.5 T for 20 representative samples. A thermomagnetic experiment was attempted using one of the most magnetized samples (at 8.97 m). The sample was crushed and dissolved in a 1 l aqueous solution of acetic acid (2% vol) for 2 h, the recovered solid product was rinsed, dried and subjected to treatment using a KLY-3 equipped with a CS-3 allowing the measurement of the temperature variations of the low field susceptibility. Even with such preparation the signal over noise ratio was poor. The low concentration of remanence bearing minerals in our samples did not allow more in depth rock magnetic investigations.

## Results and Discussion

Stable isotopes of oxygen (δ^18^O) and carbon (δ^13^C) are often use to investigate the origin and in some case to estimate the temperatures of the parental fluids^20,21^ in all our samples δ^13^C (V-PDB) show a positive values spanning from 2.7 to 6.4 while δ^18^O (V-PDB) varies from −10.2 to −7.3 (V-PDB). According to the isotopic compositions (particularly δ^13^C) our samples fall clearly into the typical class of thermogenic travertine^20,21^ (see supplementary materials).

The U concentration of our samples varies between 0.7 and 7.8 ppm; this is relatively high compared with those reported for other inorganic CaCO_3_ precipitates (speleothems, flowstones)^22,23^ or biogenic CaCO_3_ (mollusks shells, calcite corals)^2,24,25^. The ^232^Th concentration of the samples is low, varying between 0.2 and 6 ppb. As a result, detrital contamination is assumed to be negligible and no correction was applied for any of the samples. The ^234^U/^238^U activity ratio of the calcite varies from approximately 5 in samples younger than 20 ka to 1.13 in samples from the deepest part of the core. The ^230^Th/^234^U activity ratios were below secular equilibrium in all samples collected from the road section, and in samples from the upper 5.6 m of core, thus allowing ^230^Th/^234^U/^238^U ages for these samples to be calculated. The ^230^Th/^234^U/^238^U ages vary between 10 to 500 ka, depending on their stratigraphic position. Conversely, core samples from below 5.6 m have ^230^Th/^234^U values close to and/or reaching secular equilibrium, within analytical error. In a few cases for the lower part of core, particularly for samples characterized by high analytical precision (mostly those analyzed by MC-ICP-MS), we were able to calculate the finite ages associated with the large negative errors that result from subtracting the ^230^Th/^234^U analytical errors and adding the ^234^U/^238^U activity ratio analytical error due to the exponential nature of the radioactive decay phenomena. On the other hand, it was not possible to calculate the positive errors on the ages, because when the errors are summed, the samples reach secular equilibrium and the ages tend approach infinity (Table 1).

**Figure 2:**
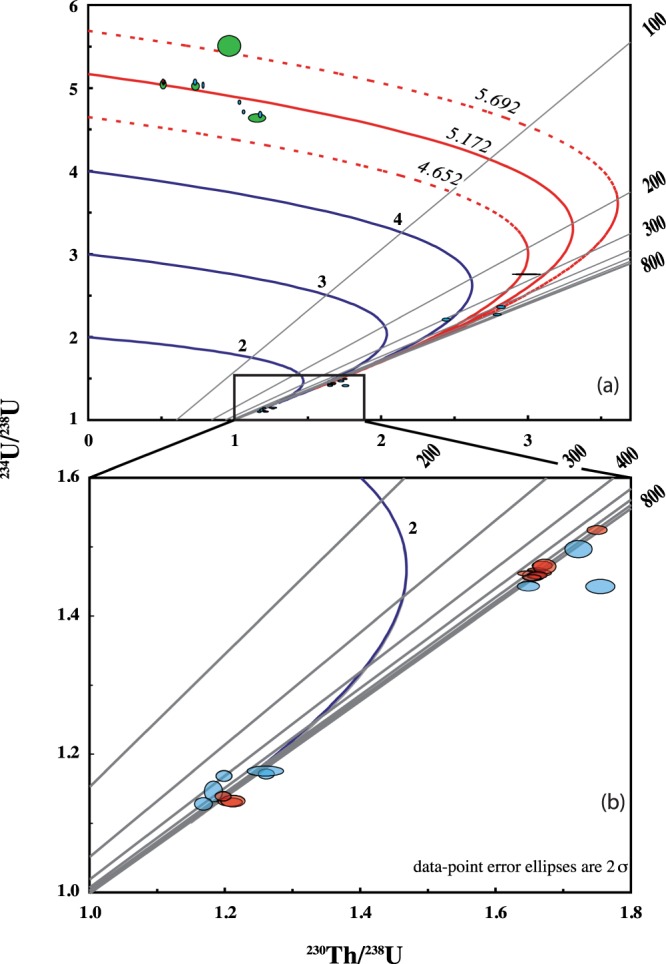
^230^Th/^238^U and ^234^U/^238^U ratios on the classical isotope evolution diagram. Light blue corresponds to TIMS, red to MC-ICP-MS, and green to alpha spectrometry data. (**b**) A close-up of the area indicated in (**a**), corresponding to the oldest samples.

**Figure 3:**
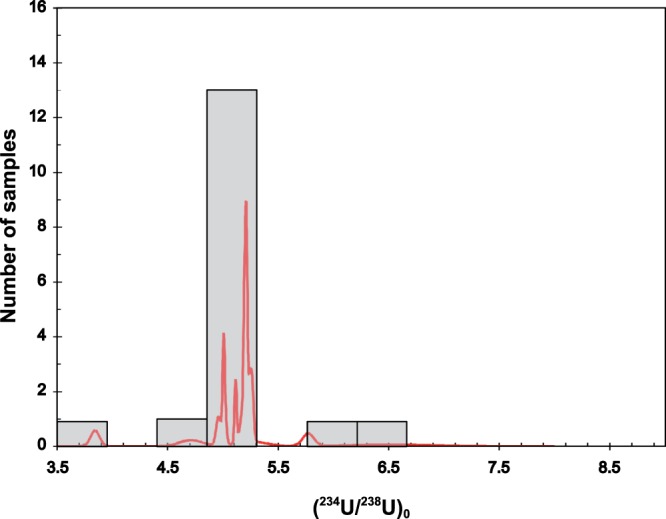
Frequency histogram of initial ^234^U/^238^U_0_ with a cumulative Gauss curve distribution for all samples yielding a finite ^230^Th/U age (17 analyses). The arithmetic mean is 5.172 ± 0.520.

Plotting the measured ^230^Th/^238^U and ^234^U/^238^U on the classical isotope evolution diagram (Fig. 2), we observe that most of samples for which finite ages were determined, from the section and the upper 5.6 m of the core, are situated on or close to the curve corresponding to the evolution of an initial ^234^U/^238^U ratio of approximately 5. The arithmetic mean of the initial ^234^U/^238^U ratio calculated for all samples yielding finite ^230^Th ages (n = 17) is 5.172 ± 0.520 (one standard deviation, Fig. 3). The high value of the initial ^234^U/^238^U_0_ corresponds to the uranium isotopic composition of the water from which the calcite was precipitated. Such high disequilibrium in water is often observed as a result of recoil effects^26,27^ and /or preferential leaching of ^234^U^28^. It is worth noting that the relative stability and high initial ^234^U/^238^U has previously been observed in hydrothermal carbonates. For example Gratier *et al*.^29^ observed a relatively constant initial ^234^U/^238^U (4.19 to 4.26) over *ca*. 1000 y in travertines from the Colorado plateau (Utah). They suggested that the constant initial ^234^U/^238^U reflects a constant fluid composition. Rhis *et al*.^30^ also found a remarkably constant initial ^234^U/^238^U over a relatively long period ~250 ka in hydrothermal carbonates from the Massif Central (France). To explain this constant initial ^234^U/^238^U Rhis *et al*.^30^ suggested the presence of deep geothermal reservoirs rocks rich in uranium that reach a steady state condition (or near steady state) with respect to water-rock interactions. In these geothermal reservoirs U is accumulated in reduced and poorly crystallized hydrothermally altered minerals that can act as a source for U to thermal water by preferential leaching and/or α recoil release. Finally another example of constant initial ^234^U/^238^U during the last 500 ka was also observed for the Devils Hole calcite vein, allowing Ludwig *et al*.^9^ to test the agreement between ^230^Th/^234^U/^238^U and ^234^U excess ages enabling him to suggest that the system had remained closed. However, recently, the closed system assumption at Devils Hole has been challenged by Moseley *et al*.^31^ because the results disagree with orbital forcing glacial-interglacial cycles. A similar mechanism can be evoked as a potential explanation to our constant initial ^234^U/^238^U. (i) The infiltration fluids reach a steady state with respect to water-rock interactions. (ii) The fluids use the same more or less pathways during infiltration (iii) The fluids show very limited variations in their physicochemical properties.

Regardless the mechanisms responsible for the nearly constant initial ^234^U/^238^U, samples located deeper in the core show two clusters: the first located between 8 and 10.1 m and the second from 10.6 to 12.6 m (Fig. 2b). For these two depth intervals, ^230^Th/^234^U/^238^U age calculations for majority of samples indicate infinite ages or a high negative error, as described above, and so we attempted to calculate the ^234^U excess ages for these two levels.

**Figure 4:**
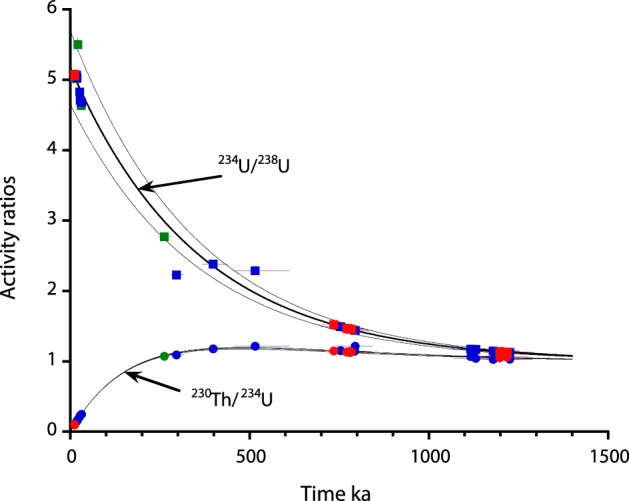
: Evolution of the activity ratios, ^234^U/^238^U and ^230^Th/^234^U, with an initial ^234^U/^238^U_0_ of 5.172 ± 0.520 and ^230^Th = 0 in closed radioactive system. Clearly samples older than 500 ky reached the asymptotic part of the ^230^Th/^234^U evolution curve while ^234^U/^238^U still indicate an easily measurable disequilibrium.

Normally, the ^230^Th/^234^U values preclude the possibility of obtaining ages older than 500 ka with reasonable errors. However, the high ^234^U/^238^U value of the initial system, 5.172 ± 0.520 (Fig. 3), makes it possible to go further back in time. For instance, if the starting point is an initial ^234^U/^238^U value of 5.172 ± 0.520, and initial ^230^Th = 0, after approximately 800 ka (as is the case for samples located between 8–10.1 m deep in the core), the measured ^230^Th/^234^U would be 1.132 ± 0.012 (uncertainty from the initial ^234^U/^238^U) and the ^234^U/^238^U value would be approximately 1.435 ± 0.054. Using these ratios to calculate ^230^Th age yields a meaningless age (with uncertainties greater than the age itself). As seen in Fig. 4 after 500 ky the ^230^Th/^234^U reached practically asymptotic part of the evolution curve, thus even small analytical errors will results in huge uncertainties on age calculations. On the other hand the ^234^U/^238^U activity ratios still indicate value with an easily measurable disequilibrium value, thus allowing an age to be calculated based on the decay of ^234^U excess (Fig. 4).

Assuming that samples from deeper than 5.6 m in the core were precipitated from an aqueous phase similar to the upper part of the core, with an initial ^234^U/^238^U of 5.172 ± 0.520, ^234^U excess ages can be calculated for the lower part of the core using the following equation^32^:

$${({}^{234}{\rm{U}}{/}^{238}{\rm{U}})}_{{\rm{t}}}=1+([{}^{234}{\rm{U}}{/}^{238}{{\rm{U}}}_{0}-1]\ast {{\rm{e}}}^{\mbox{--}\lambda 234\ast {\rm{t}}})$$

where (^234^U/^238^U)_0_ is the calculated initial ^234^U/^238^U activity ratio; ^234^U/^238^U_t_ is the measured ^234^U/^238^U activity ratio; λ_234_ is the U decay constant, ^234^U; and t is the ^234^U excess age.

**Figure 5:**
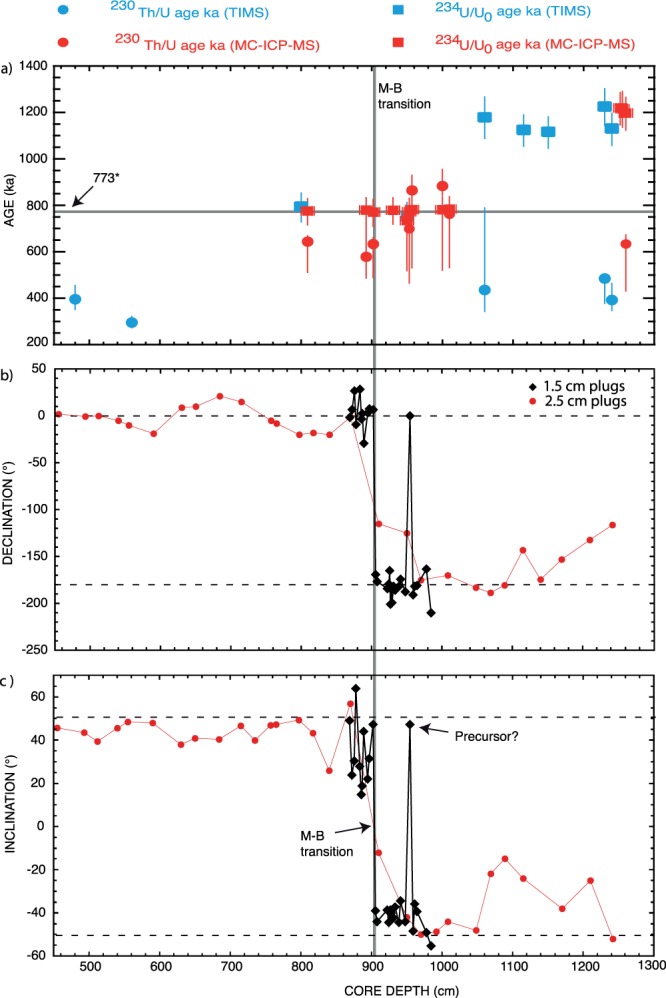
Ages and paleomagnetic results vs depth, the vertical grey line marks the Brunhes-Matuyama transition determined after the paleomagnetic results (**a**) Isotopic ratios and ages determined using the standard ^230^Th/U approach and the new ^234^U/U_0_ ages, horizontal grey line is mean age for B-M obtained by Channel *et al*.^36^ (773 ka). (**b**) Paleomagnetic declinations, red circles are data from the 2.5 cm plugs, black diamonds from the 1.5 cm plugs, the core is reoriented so that the mean declination is along the N-S direction, dashed lines are the 0 and 180° expected dipole values. (**c**) same than (**b**) for paleomagnetic inclinations, dashed lines are the + 51° and −51° expected dipole value.

Using the above equation, we calculated ^234^U excess ages for all samples from deeper than 5.6 m in the core (Table 1). The maximum errors associated with these age calculations were determined by combining the highest initial ^234^U/^238^U value + 1 standard deviation with the lowest measured ^234^U/^238^U value – 2 σ, and vice versa. The ^234^U excess ages in the present case allow the calculation of older ages than would be possible through the usual U-series dating (~500 ka). Figure 4 shows the evolution of the activity ratios, ^234^U/^238^U and ^230^Th/^234^U, with an initial ^234^U/^238^U value of 5.172 ± 0.520 in a closed radioactive system. As seen in Fig. 5, when the calculated ^230^Th/^234^U/^238^U and ^234^U excess ages are plotted as a function of depth in the core, samples located between 8 to 10.1 m and 10.6 to 12.6 m depth yield ages clearly clustered into two periods of time; the first, 776 ± 14 ka, corresponds to an at least 2.1 m-thick travertine unit, and the second, 1173 ± 22 ka, corresponds to an approximately 2 m-thick unit. The results also show that travertine accretions seem to correspond to pulses of hydrothermal activity, which allows the precipitation and accumulation of thick CaCO_3_ layers over relatively short time intervals.

Fifty eight samples (28 1.5 cm plugs and 30 2.5 cm plugs) provided demagnetization paths with convergence towards the origin allowing to determine characteristic remanent magnetization (ChRM) directions (Fig. S1-a). The other samples have erratic behavior mostly associated to poor signal/noise ratio (with bulk magnetization sometimes on the order of 10^−10^ Am^2^) or gyromagnetic remanence. The demagnetization loss at 70 mT is between 95 to only 60% in few cases. IRM experiments up to 2.5 Tesla confirm that the remanent magnetization in all samples is dominated by a low coercivity phase, in addition to a high coercivity phase (characterized by magnetization acquired above 300 mT) in variable proportion (Fig. S1-b). The low coercivity phase is interpreted as being magnetite, the high coercivity phase hematite and/or goethite in accordance with previous results from travertine (e.g. Piper *et al*.^33^; Lebatard *et al*.^34^). The thermomagnetic curve from the sample at 8.97 m (Fig. S1-c) depicts a mere hump until 400 °C which is compatible with magnetite but a transformation starting at 450 °C and attributed to the decomposition of siderite into magnetite (Pan *et al*.^35^), did not allow to characterized further the magnetic mineralogy.

In spite of the difficulty in characterizing further the magnetic mineralogy, the low coercivity phase is the dominant remanence carrier in all samples and straight line fits of the ChRM to the origin indicates that, when present, high coercivity phases do not carry a different direction of magnetization, giving confidence in the results. Resultant paleomagnetic inclinations and declinations after demagnetization are shown in Fig. 5. Throughout the section the inclination changes from reverse direction with a mean of −42° to normal direction with a mean of 43°. The inclination record is shallower but overall consistent with the current expected geocentric axial dipole field inclination for the location from which the core was sampled (51°). Declination is rotating by 180° at a level coincident with the change in the sign of inclination. The dense sampling between 8.49 and 9.86 m allows to precisely localized the change in direction at around 9.04 m where low NRM did not allow to isolate a ChRM. One of the small plug at depth of 9.55 m is giving a normal polarity direction, this may be a true geomagnetic event but we cannot ruled out the occurrence of a restrained remagnetization event. The magnetic polarity sequence data allows the inversion at ~9.04 m to be attributed to the Brunhes-Matuyama (B-M) transition. The latest mean age suggested for this reversal is 773.1 ka^36^. The average weighted age obtained here through the ^234^U excess dating approach (776 ± 14) is in excellent agreement with previous results using other methods^1^.

## Conclusions

The combination of ^230^Th/U dating with the ^234^U excess approach applied to a travertine sequence in Morocco allows U-series dating to be extended to approximately 1.2 million years, if the assumption of a constant initial ^234^U/^238^U is valid. In the present study, a time constraint on the Brunhes-Matuyama magnetic polarity inversion is obtained through this approach using U-Series (776 ± 14 ka) and is in good agreement with the age of the inversion previously obtained using other dating methods, such as K-Ar and ^40^Ar/^39^Ar argon family methods^37,38^. According to the best of our knowledge, this is the first time that the B-M reversal has been radiometrically dated using the U-series approach applied to continental calcium carbonates.

## Supplementary information

Supplementary information
