# Supplementary material for: Timing of the Brunhes-Matuyama transition constrained by U-series disequilibrium
Source: Sci Rep. 2019 Apr 15;9:6039. doi: 10.1038/s41598-019-42567-2 (PMC6465598; doi:10.1038/s41598-019-42567-2)
Supplement: Supplementary file 1 — Supplementary information [file 41598_2019_42567_MOESM1_ESM.pdf]

Timing of the Brunhes-Matuyama transition constrained by U-series disequilibrium

Supplementary information

Bassam Ghaleb[1*], Christophe Falguères[2], Julie Carlut[3, 4], Jean-Pierre Pozzi[3, 2], Geoffroy Mahieux[5], Larbi Boudad[6], Louis Rousseau[2]

Isotopic methods

All the analyses were performed in the SSMIM (Service de Spectrométrie Isotopique du Muséum) of the Muséum national d'histoire naturelle using a Kiel IV (Thermo) automatic device for carbonate preparation, which is linked to an IRMS Delta V Advantage (Thermo) mass spectrometer. Samples of between 29 to 68 µg of carbonate powder were reacted with pure orthophosphoric acid (H_3PO_4) at 70°C in individual reactors. The CO_2 produced was purified by cryogeny. The uncertainty of this analytical measurement process is assessed by analyzing 6 samples by an internal standard (LM marble normalised by NBS18). This uncertainty is 0,022 ‰ (k=1) for $d^{13}C$, and 0,019 ‰ (k=1) for $d^{18}O$. Other analyses were performed at Laboratory of Geography of Ottawa, following the same protocol, using a Gas Bench II device coupled to a Finnigan Mat Delta+ XP mass spectrometer.

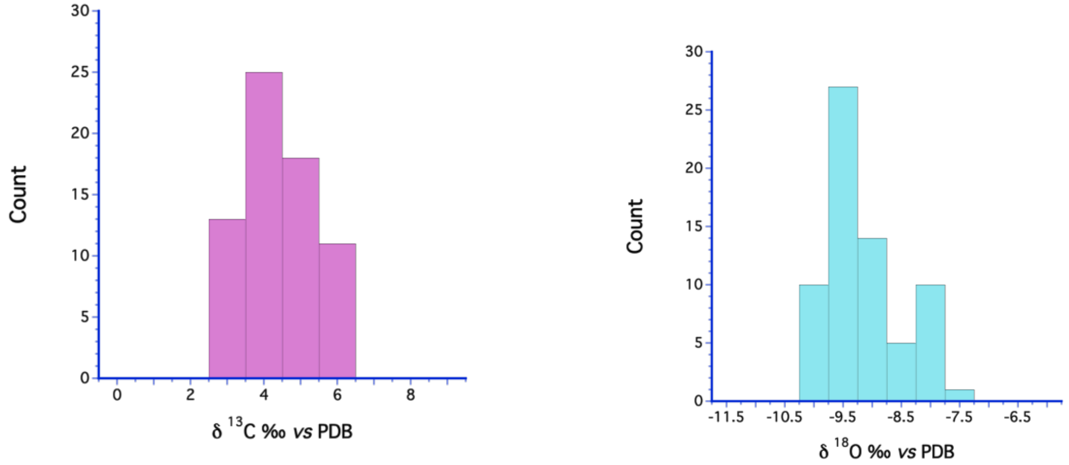

Figure 1 SP: stable isotopes $\delta^{13}C$ & $\delta^{18}O$ histogram frequency for travertine samples from the IRDI core. According to classification described by Pentecost 2010, our stable isotopes result indicates a thermogenic origin of the travertine.

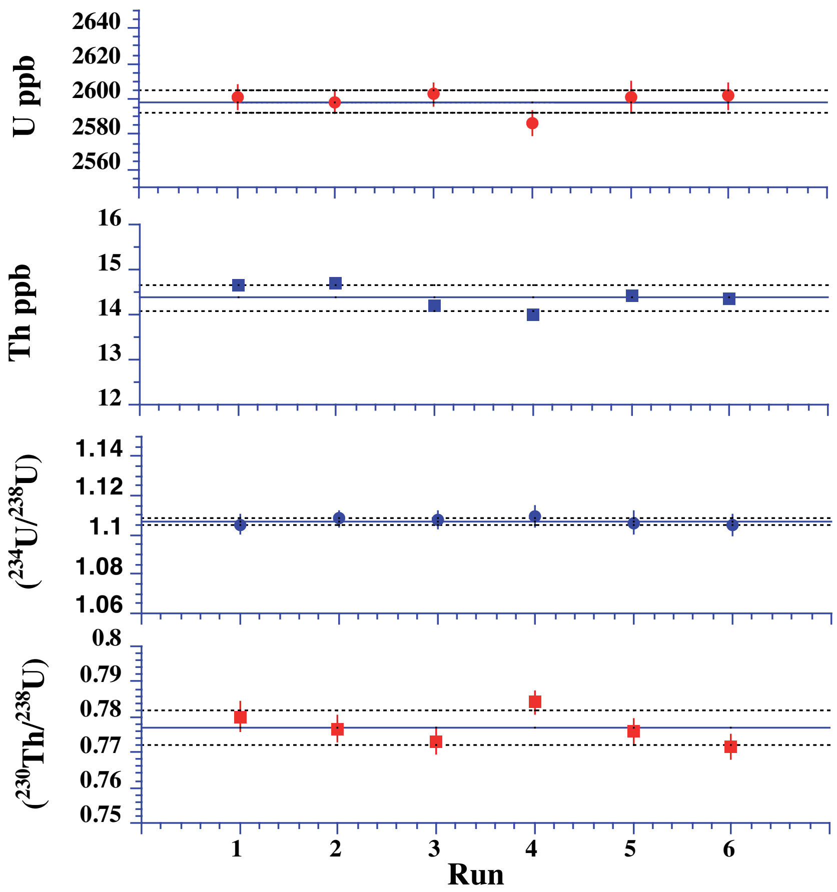

Figure 2 SP: reproducibility of [U], [Th], ($^{234}U/^{238}U$) and ($^{230}Th/^{238}U$) on coral samples from last interglacial period from Cabo Verde. Instrumental reproducibility is 0.25%, 2 %, 0.17% and 0.65% for [U], [Th], ($^{234}U/^{238}U$) and ($^{230}Th/^{238}U$) respectively.

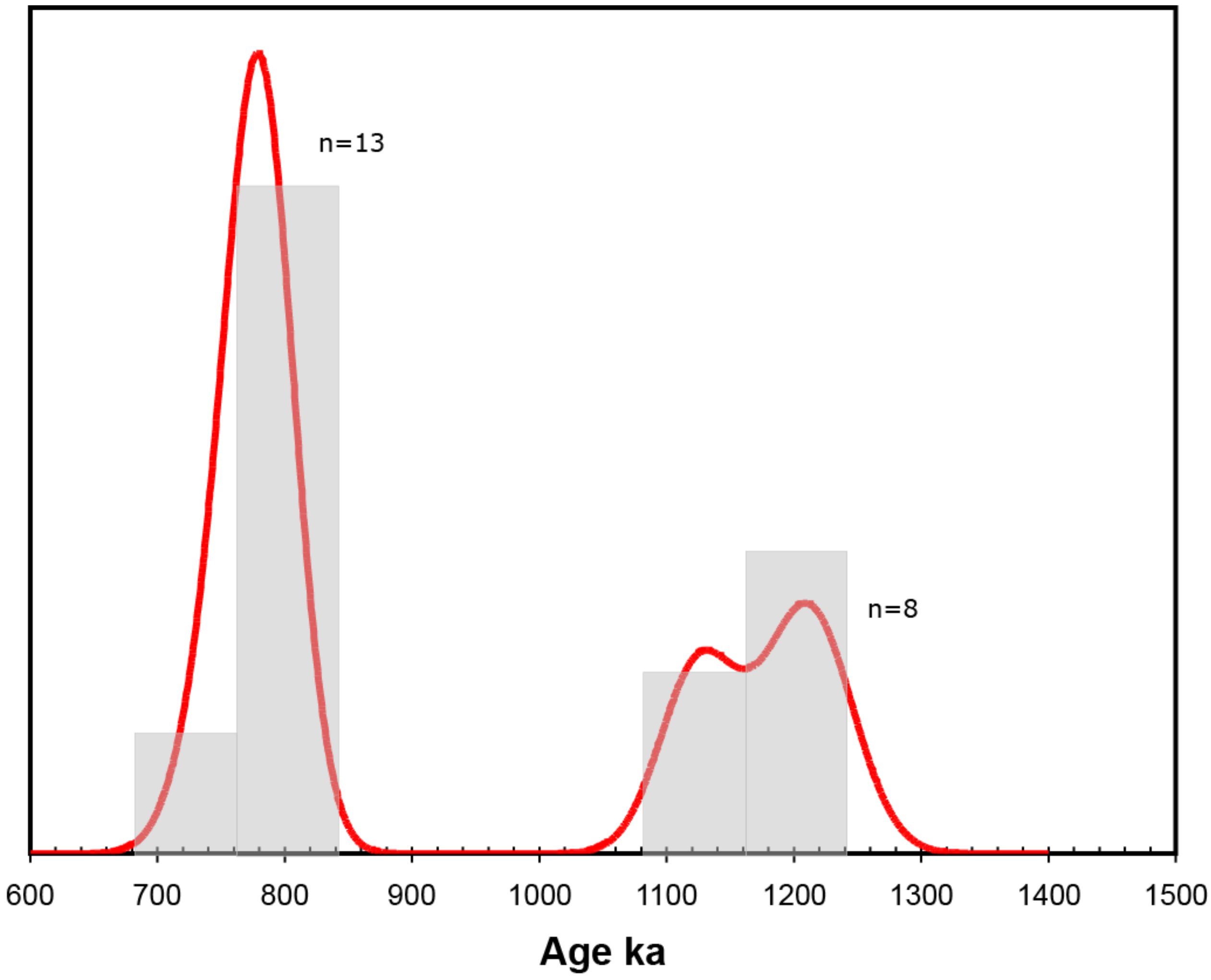

Figure 3 SP: Cumulative Gauss curve for ages obtained by ^{234}U excess dating. The figure shows two clusters of ages, the first one is 776 ± 14 ka (n=13) and the second 1173 ± 22 ka (n=8). Given the thickness of travertine in the core corresponding to these two periods, we conclude that accretion of travertine is relatively fast and could correspond to a pulse of hydrothermal activity.

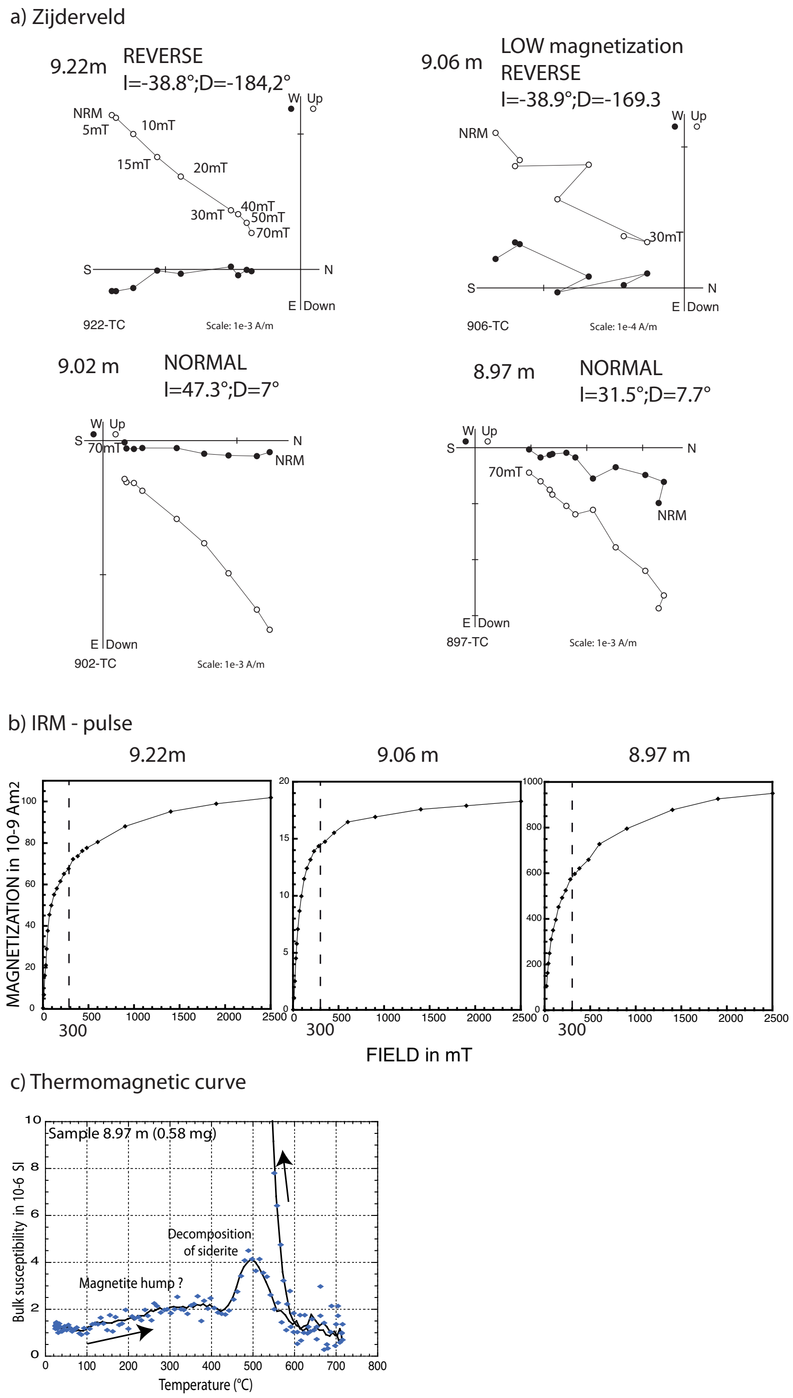

Figure 4 SP: Zijderveld demagnetization diagrams for 4 small plug samples. IRM acquisition curves showing a mixture of low and high coercivity phases.

References

Pentecost, A., 2010. Travertines, Spinger Edit. 445 pp.
